# Recent Developments in Fiber Optics Humidity Sensors

**DOI:** 10.3390/s17040893

**Published:** 2017-04-19

**Authors:** Joaquin Ascorbe, Jesus M. Corres, Francisco J. Arregui, Ignacio R. Matias

**Affiliations:** 1 Department of Electrical and Electronic Engineering, Public University of Navarra, Pamplona 31006, Spain; jmcorres@unavarra.es (J.M.C.); parregui@unavarra.es (F.J.A.); natxo@unavarra.es (I.R.M.); 2 Institute of Smart Cities, Public University of Navarra, Pamplona 31006, Spain

**Keywords:** optical fiber humidity sensors, fiber Bragg gratings, long period fiber grating, photonic crystal fiber, tapered optical fiber, modal interferometer, Fabry-Pérot interferometers, resonators, lossy mode resonances

## Abstract

A wide range of applications such as health, human comfort, agriculture, food processing and storage, and electronic manufacturing, among others, require fast and accurate measurement of humidity. Sensors based on optical fibers present several advantages over electronic sensors and great research efforts have been made in recent years in this field. The present paper reports the current trends of optical fiber humidity sensors. The evolution of optical structures developed towards humidity sensing, as well as the novel materials used for this purpose, will be analyzed. Well-known optical structures, such as long-period fiber gratings or fiber Bragg gratings, are still being studied towards an enhancement of their sensitivity. Sensors based on lossy mode resonances constitute a platform that combines high sensitivity with low complexity, both in terms of their fabrication process and the equipment required. Novel structures, such as resonators, are being studied in order to improve the resolution of humidity sensors. Moreover, recent research on polymer optical fibers suggests that the sensitivity of this kind of sensor has not yet reached its limit. Therefore, there is still room for improvement in terms of sensitivity and resolution.

## 1. Introduction

Humidity has an important influence on several industrial processes such as electronic, food or pharmaceutical manufacturing, food storage, etc. All these processes, which can be affected by humidity, require continuous monitoring of air humidity. In addition, proper humidity levels can be critical to the quality of the product and having the right humidity level can contribute to diminishing energy consumption [1]. 

Optical fiber humidity sensors (OFHSs) offer several advantages over electronic humidity sensors such as miniature design, durability, the possibility of working on flammable environments and at higher temperature and pressure ranges, and, most important, their electromagnetic immunity. Therefore, they can withstand the kind of harsh and demanding conditions found in industrial processes. 

However, there are some facts that have prevented OFHSs from being a common commercial product. The fabrication of optical fiber sensors is not yet a sufficiently repeatable process to become a serial fabrication product. Some optical structures have a certain degree of uncertainty inherent to the fabrication process. However, the main inconvenience is related to the cost of optical equipment. Halogen white light sources and standard optical fibers can be found on the market at affordable prices, but spectrometers, optical spectrum analyzers (OSA), and other optical equipment have relegated OFHSs to specific applications where there is no other option, as detailed below.

Great research efforts have been made in recent years and results have been generated for various applications. The accurate measurement of relative humidity (RH) is critical in some industries, such as the semiconductor industry, where the performance of fabricated devices is dependent on the humidity [2], as well as in the electronics industry. Recently, another application for these OFHSs has been found in high-energy physics (HEP), in experiments performed at the European Organization for Nuclear Research (CERN) [3,4].

More application fields can be found, some of them related to structural health monitoring, which is an important case where relative humidity (RH) sensors can find application [5,6,7,8]. OFHSs offer the possibility of monitoring civil engineering structures, using, for instance, fiber Bragg gratings (FBG) [6,7], or even obtaining distributed measurements for large structures combining Optical Time Domain Reflectometry (OTDR) with chemically sensitive water swellable polymers (hydrogels) [9]. In addition, the FBG sensors mentioned above have the potential to act as road parameter sensors (humidity, ice, temperature, etc.) [7]. Another application found for distributed measurement is for tunnel leakage detection [10]. The possibility of making distributed measurements is one of the strongest advantages of OFHS [11,12]. Another sector where this kind of sensor is useful is food processing and storage [6]. Furthermore, humidity control is essential for the correct storage of valuable artwork, such as in museums or archives [2]. 

Apart from the aforementioned applications, continuous RH measurement and control is important for human comfort such as in air-conditioning monitoring and achieving controlled hygienic conditions [6]. In addition, OFHSs have found new applications in clinical treatment due to the need for humidification of inspired gases in critical respiratory care [13,14].

The present paper reports the current trends of optical fiber humidity sensors. Novel optical structures, as well as recently studied materials, will be analyzed and commented on. Then, a detailed analysis of OFHSs based on lossy mode resonances will be made. This kind of sensor occupies a unique position alongside optical fiber sensors due to its relatively simple fabrication process and high sensitivity to the surrounding medium refractive index (SMRI). Finally, some concluding remarks about the sensing performance of all these sensors will be expounded.

## 2. Recent Trends in Optical Fiber Humidity Sensors 

**Figure 1:**
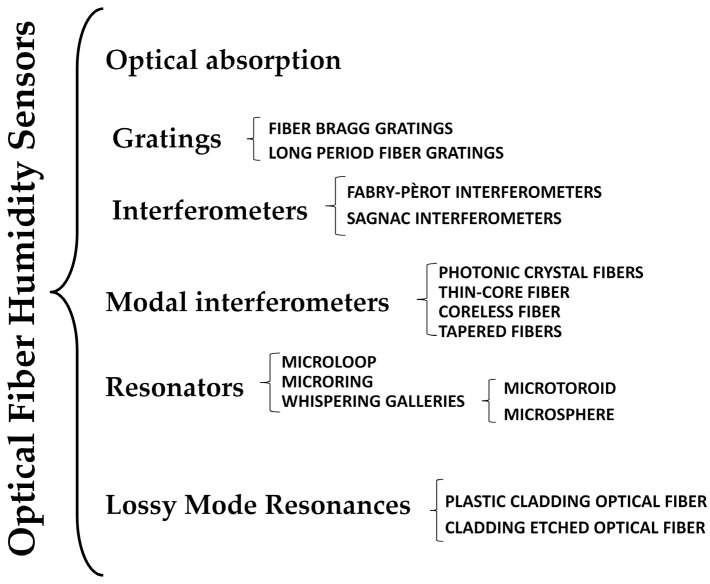
Classification of optical fiber humidity sensors.

Here, a brief review of the most recently developed OFHSs will be presented. The OFHS have been classified according to their working principle. The first group includes OFHS based on the optical absorption of materials, which were the first kind of OFHS developed. The next group includes OFHS based on fiber Bragg gratings (FBG) and long-period fiber gratings (LPFG). Another possibility for the development of OFHS is based on interference, which can be divided into several kind of interferometers such as Fabry-Pérot, Sagnac, Mach-Zehnder, Michelson, and modal interferometers. The next category includes OFHS based on micro-tapers, micro-ring, and micro-knot resonators (MKR), as well as other sensors based on whispering galleries modes (WGM). Finally, OFHS based on electromagnetic resonances, specifically lossy mode resonances (LMRs), will be discussed. A scheme of the proposed classification is shown in Figure 1.

### 2.1. Optical Absorption Sensors 

These sensors are based on the interaction of the evanescent field with the coating used as the sensitive material, providing changes of the transmitted optical power along the whole spectrum. For this kind of sensor, plastic-cladding silica (PCS) optical fiber or plastic optical fibers (POF) are commonly used, although there are other possibilities such as the side-polished optical fiber (D-shape). Some of these optical fibers present advantages such as low fabrication cost [15], the possibility of measuring with a simple setup, and high reliability, but they have some disadvantages. The main disadvantages are related to the method of measurement, which only detects changes on the transmitted optical power; these changes might be affected by undesired factors such as fluctuations in the light source. 

Most recent research has focused on the study of materials that are becoming common in the development of optical fiber sensors. These materials are being studied for their ability to improve certain characteristics of OFHS, such as response time or sensitivity. The studied materials are tungsten disulfide [16], reduced graphene oxide (rGO) [17], and zinc oxide [18]. 

**Figure 2:**
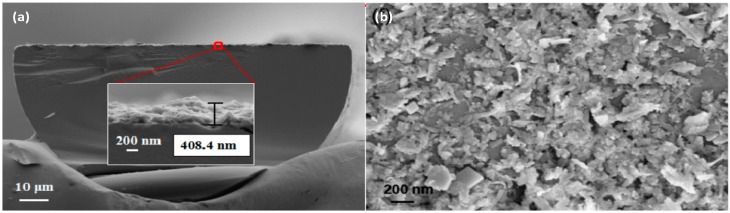
(**a**) D-shape optical fiber coated with tungsten disulfide coating and (**b**) SEM image of the surface of the coating at a magnification of 31270. Reprinted from [16] with permission from OSA Publishing.

Tungsten disulfide (WS_2_) has been studied because the physical adsorption of the water molecules onto the WS_2_ layer is accompanied by a moderate degree of charge transfer, enabling fast response times [16]. A D-shape optical fiber was coated with WS_2_, as can be seen in Figure 2, and this OFHS has shown a sensitivity of 0.1213 dB/%RH and a resolution of 0.475%RH. The rising time is about 1 s and the recovery time is about 5 s. 

The sensor developed with rGO has focused on achieving high sensitivity for high humidity values. It displays a linear response in the 70%–95%RH range, a sensitivity of 0.31 dB/%RH, and its response speed is faster than 0.13%RH/s. 

On the other hand, there are certain materials that provide high sensitivity in extremely low-humidity environments [19], which is very useful in applications such as lithium-ion battery manufacturing, semiconductor fabrication, archival storage and preservation, and the pharmaceutical industry. This OFHS was fabricated using a U-bent optical fiber coated with silica film by sol–gel process and doped with methylene blue. For RH ranging from 1.1% to 4.1%, the sensor shows a linear relationship, with a sensitivity of 0.087 dB/%RH and a limit of detection of 0.062%RH [19]. The response and recovery time of the sensor is 20 s–3 min depending on the RH variation. 

**Figure 3:**
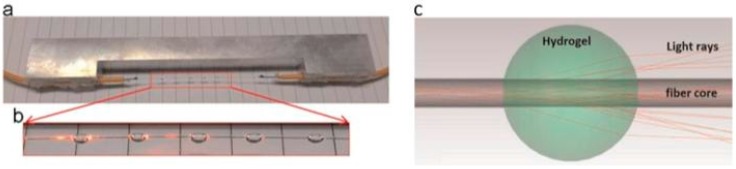
Photo of a fabricated sensor with five hydrogel spheres, attached on a section of fiber core. (**a**) The whole sensor; (**b**) a detailed view of five spheres; and (**c**) an illustration of the light rays that travel through a single hydrogel sphere with a diameter of 2 mm and a refractive index of 1.45. Reprinted from [20] with permission from Elsevier.

With regard to novel structures, the use of 2-mm hydrogel spheres for humidity detection should be mentioned [20]. Rather than coating the bare optical fiber core with a coating of even thickness, the reported sensor exploits hydrogel spheres on the fiber core (see Figure 3). Nevertheless, the sensing range of the current sensor is still very narrow, from 70% to 90%, due to the moisture absorption behavior of the hydrogel used.

### 2.2. Fiber Bragg Gratings

A Bragg grating is an optical structure that consists of a periodic perturbation of the refractive index of a waveguide. A FBG is formed by exposure of the core of the optical fiber to an intense optical interference pattern of ultraviolet light [21]. The exposure produces a permanent increase in the refractive index of the core of the fiber, creating a fixed index modulation according to the exposure pattern [22]. When light is launched into the optical fiber, a small amount of light is reflected at each periodic refraction change. All the reflected light signals combine coherently into one large reflection at a particular wavelength when the grating period is approximately half of the input light’s wavelength [22]. The grating does not affect light propagating at wavelengths different from the Bragg wavelength, which satisfies Equation (1):

(1) $$\lambda_{B}=2n\Lambda ,$$

where *λ_B_* is the Bragg wavelength, *n* is the effective refractive index of the grating in the fiber core, and *Λ* is the grating period. Due to the mask dimensions and the characteristics of standard communications single mode optical fibers (SMF), Bragg wavelength is usually located at the infrared range. More recent research involves the use of micro-structured [23] plastic optical fibers (POFs) or SMF with reduced core diameter to be able to work in the visible-near-infrared range [24].

Hygroscopic materials are commonly used to develop OFHSs using FBGs because of the strain they can apply to the FBG when swelling [25]. A large number of research papers [25,26,27,28,29,30,31,32,33] have been published recently dealing with this optical structure and, despite its low dynamical range and sensitivity, FBGs still attract great interest due to their inherent multiplexing capability and high quality [33]. Moreover, etching the cladding of a FBG and coating it with a sensitive layer enhances the sensitivity of this optical structure [25]. 

Several polymeric materials have been coated onto a FBG and tested for humidity sensing purposes such as polyimide [4,31], di-ureasil, [34] or poly(methyl methacrylate) (PMMA) [25,28], among others. The sensitivity of FBGs developed in [4], coated with polyimide by dip-coating, range from 1.4 to 5.6 pm/%RH depending on the thickness of the coating. However, the same material has provided greater sensitivities (13.6 pm/%RH) when coated by another method (in situ imidization) [31]. According to the results of both papers, the sensitivity increases with the thickness of the coating. When compared with polymer-based solutions, the proposed di-ureasil layer shows an enhanced sensitivity of 22.2 pm/%RH [34].

**Figure 4:**
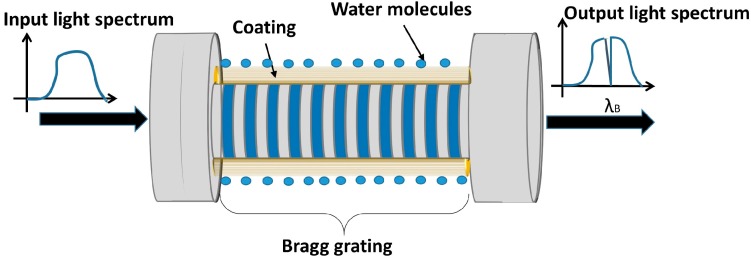
Schematic of an optical fiber humidity sensor using fiber Bragg gratings.

Graphene oxide [32] and carbon nanotubes (CNT) [33] have also been tested as the sensitive layer for FBG-based RH sensors. CNTs, coated onto an etched FBG, provide the highest sensitivity found for conventional FBGs OFHS, which is 31 pm/%RH [33]. A representation of this optical device can be seen in Figure 4.

Concerning the cross thermal sensitivity, which is inherent to FBGs, it has been compensated for in [30] by inscribing the grating on a High-Birefringent (Hi-Bi) optical fiber. Due to the birefringence of the fiber, the FBG exhibits a reflectivity spectrum dominated by two separated Bragg wavelengths, whose separation depends only on the temperature. 

**Figure 5:**
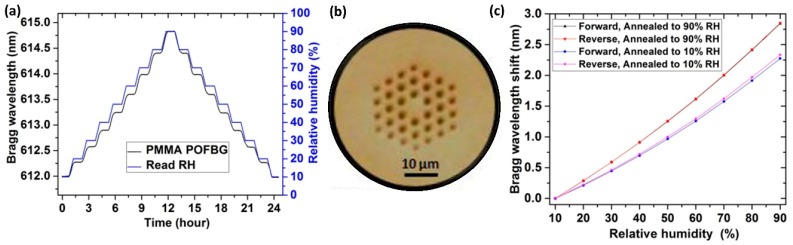
(**a**) Measured humidity response at 25 °C of PMMA mPOFBG annealed up to 90%RH versus time and humidity; (**b**) Microscope image of the end facet of PMMA mPOF; (**c**) Corresponding stabilized response of the PMMA mPOFBGs annealed up to 90% and 10%. Reprinted from [27] with permission from OSA Publishing.

Finally, recent research has focused on improving the sensitivity by using PMMA-based micro-structured polymer optical fiber Bragg gratings (POFBG) [27]. This structure has been demonstrated to have a superior response with very low hysteresis, improved sensitivity (35 pm/%RH), and an increased stable operation temperature if it is annealed at high humidity (90%RH). Some results related to this optical device are shown in Figure 5.

### 2.3. Long-Period Fiber Gratings

Long-period fiber gratings (LPG) consist of a periodic modification of the refractive index of the core of a single-mode optical fiber (SMF). In opposition to FBG, which have a sub-micron period and couple light from the forward-propagating mode of the optical fiber to a backward counter-propagating mode, LPGs have a period typically in the range of 100 µm to 1 mm. This provokes in LPGs a coupling of light between the guided core mode and various co-propagating cladding modes [35,36]. This coupling produces a series of attenuation bands in the optical fiber transmission spectrum, each one centered at a different resonant wavelength. A portion of the electromagnetic field of the cladding modes penetrates the surrounding medium in the form of an evanescent wave [37]. Although LPGs were initially developed as rejection-band filters [38], they also present interesting characteristics for sensing.

**Figure 6:**
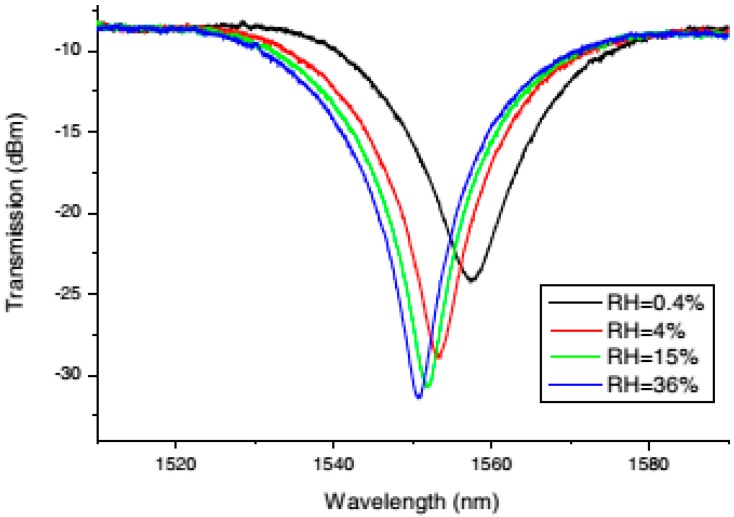
Transmittance spectra of LPG acquired at different RH values. Reprinted from [37] with permission from OSA Publishing.

Several kinds of materials have been explored as coatings for the development of LPG-based RH sensors, including polymers [39,40], hydrogels [41], gelatin [42], cobalt-chloride-based materials [43], and SiO_2_ nanospheres [44]. Most recent research has focused on studying the performance of this sensor for low values of RH (0.4% to 36%RH) and temperature (−10 to 20°C) and subjected to radiation [37]. For this purpose, the material chosen for the coating was titanium dioxide and the sensitivity obtained was 1.4 nm/%RH at low RH values, which is a high sensitivity for a LPG. Measured spectra of device developed in [37] are shown in Figure 6.

**Figure 7:**
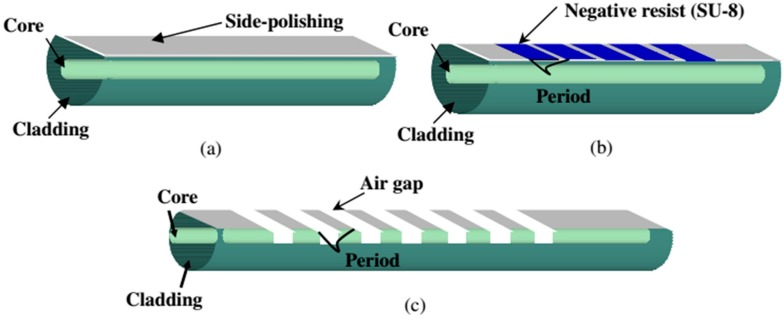
Fabrication process of the air gap LPG. Reprinted from [45] with permission from Optical Society of Japan.

A novel method for the fabrication of LPGs (schematized in Figure 7) has been developed in [45]. It combines the fiber side-polishing and fiber etching methods to create air gaps on the polished region that reach the core of the optical fiber. After coating this device with calcium chloride, we obtained a sensitivity as high as 1.36 nm/%RH in the range 55%–90%RH.

Another novel approach for the development of OFHS based on LPG was studied in [46]. First, a LPG was coated by layer-by-layer nano-assembly (LbL) method with PAH/PAA. Then, by chemically removing half of the LPG coating, the main attenuation band was split into two different contributions. The coated LPG contribution remained and a second band appeared because of the removing process. As was expected, this new band corresponds to the half-uncoated LPG area. When this semi-coated LPG was also exposed to RH and temperature tests, the two new attenuation bands presented different behaviors for humidity and temperature. The dual-wavelength-based measurement provided a simultaneous monitoring of RH and temperature, with sensitivity ratios of 63.23 pm/%RH and 410.66 pm/°C for the attenuation band corresponding to the coated contribution, and 55.22 pm/%RH and 405.09 pm/°C for the attenuation band corresponding to the uncoated grating.

### 2.4. Modal Interferometers

Fiber optics technology offers many degrees of freedom for the generation of modal interferometers. Therefore, several structures have been studied, all of them providing advantages such as stability, compactness, small size, lightness, etc. [47,48,49]. The interferometric phase difference is built up by considering the difference in the effective refractive indices of different fiber modes [50]. There are alternative topological configurations such as Michelson or Mach-Zehnder interferometers, which can be implemented by means of splicing different types of fibers in a hybrid structure [51] or by other methods [52].

#### 2.4.1. Photonic Crystal Fibers

**Figure 8:**
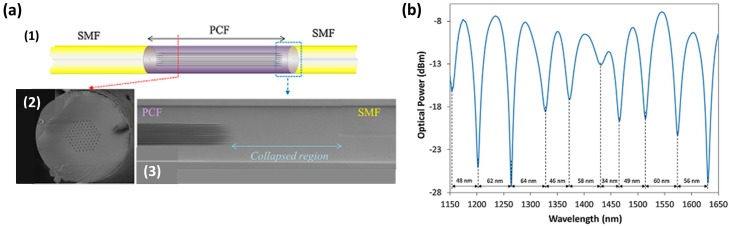
(**a**): (**1**) Schematic representation of the photonic crystal fiber interferometer; (**2**) cross section of the PCF used to build up the interferometer; (**3**) micrograph of the PCF-SMF junction showing the collapsed region; (**b**) Transmission spectrum of the PCF-I. Reprinted from [56] with permission from Elsevier.

Photonic crystal fibers (PCF) can be included as Mach-Zehnder interferometers (MZI), although they can also be used as Michelson or Sagnac interferometers [53]. These fibers are characterized by a complex pattern of microscopic air-holes in the transverse plane that runs all along the fiber [53,54,55] . PCFs are attracting great interest due to the different alternatives to construct all-fiber modal interferometers such as tapered PCF or hybrid structures [53]. The key element in these interferometers is a microscopic region in which the voids of the PCF are fully collapsed [53]. The two collapsed interfaces between PCF and SMF segments produce the excitation and recombination of core and cladding modes [56]. A scheme of the optical structure and the obtained transmitted spectrum can be observed in Figure 8.

In [57] it is reported that a coating is not needed with these interferometers to obtain a temperature-insensitive OFHS. Coating the PCF with hygroscopic materials such as agarose [58,59], polyvinyl alcohol (PVA) [60], or poly(allylamine hydrochloride) (PAH) and poly(acrylic acid) (PAA) [56] improves the sensitivity of these interferometers, reaching 2.35 nm/%RH in the range 75%–95%RH. However, these materials present non-linear behavior, although linearization can be achieved by using a digital processing algorithm [56].

#### 2.4.2. Tapered Optical Fibers

As previously mentioned, MZI require two different optical paths for generating interference. One optical path is the core of the optical fiber, while the other optical path is the cladding, where the cladding modes are guided through. For this reason, it is necessary to allow the cladding modes to propagate through the cladding to obtain a modal interferometer based on MZI. Several approaches have been followed for this purpose.

One of the most well-known structures is the non-adiabatic tapered optical fibers (NATOF). In NATOFs the fundamental mode is coupled to higher order modes, which generate modal interference and, therefore, an oscillatory optical power output [61,62,63]. In a tapered SMF, the central region of the taper acts as a multimode fiber and the light is now guided through the cladding of the fiber, which makes the surrounding medium play the role of the new cladding [61]. This coupling of modes in the taper waist makes the taper very sensitive to surrounding medium refractive index (SMRI) changes, allowing its use as an OFHS by adding an appropriate coating. The silica taper developed in [64], with a waist diameter of 3.8 µm, has demonstrated RH sensitivity of 97.76 pm/%RH with a cross thermal sensitivity of only −0.048%RH/°C, without any extra functional coatings. In a similar way, the micro-wires utilized in [65,66] do not require additional coatings for obtaining an OFHS, providing sensitivities of 114.7pm/%RH in the 30%–90%RH range [65] and 0.14 rad/%RH in the 20%–70%RH range [66]. 

**Figure 9:**
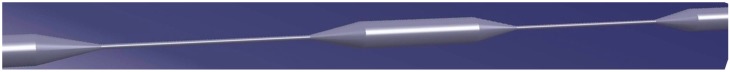
Scheme of a double in-line tapered optical fiber conforming to a modal Mach-Zehnder interferometer.

Double in-line adiabatically tapered optical fibers (see Figure 9) can also be considered a modal MZI [67]. The first tapered region diffracts the fundamental mode and consequently allows the cladding modes to become excited. The differences between the effective refractive indices of the core and cladding modes result in phase shifting. Increasing the RH affects the effective RI of the cladding modes, with the RI of the core mode remaining unmodified. Based on that working principle, sensitivities of 20 pm/%RH were obtained, without any sensitive layer. The main disadvantage is its high temperature sensitivity.

#### 2.4.3. Modal Interferometers Obtained by Different Combinations of Fibers

Another method to obtain a MZI is by splicing a standard SMF to a thin-core optical fiber, as was studied in [29]. A fraction of the light that was being guided through the core of the SMF is guided through the cladding of the thin-core fiber. Therefore, light has two different optical paths and the interferometer is generated. Furthermore, a FBG was written onto the thin-core section, which was also coated by Layer-by-Layer nano-assembly (LbL), so simultaneous measurement of temperature and RH can be accomplished. The estimated resolution of the sensor is 0.78%RH.

**Figure 10:**
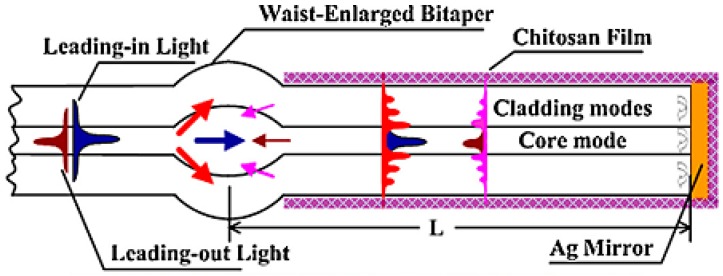
Schematic diagram of the proposed Michelson interferometer-based humidity sensor. Reprinted from [69] with permission from Elsevier.

More approaches have been followed in the last few years to advance modal interferometry. In [51], the MZI is constructed of two waist-enlarged tapers and other explored options are one waist-enlarged taper followed by an offset [68]. These optical structures provide a good interference pattern and changes on the transmitted optical power of 0.35 dB/%RH [51]. Recently developed Michelson interferometers are quite similar to previous MZI, with the difference of light going through each optical path twice, as schematized in Figure 10. Therefore, similar structures were used for the development of these interferometers, which provides sensitivities of 135 pm/%RH [69].

**Figure 11:**
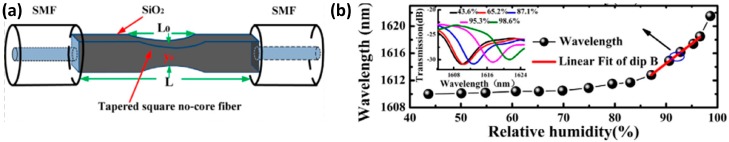
(**a**) Schematic diagram of the proposed Michelson interferometer-based humidity sensor; (**b**) Transmission spectral responses of dip B under different environmental RH levels. Reprinted from [72] with permission from Elsevier.

The single mode-multimode-single mode (SMS) fiber structure has found many applications owing to its unique spectral characteristics [70]. The physical principle of this interferometer is that light transmitted through the fundamental mode of the SMF is coupled to several modes into the MMF section and re-coupled to the fundamental mode of the SMF at the end of the MMF segment [70]. A SMS optical fiber coated with PVA provides a sensitivity of 90 pm/%RH [71], whereas by combining SMS optical structure, the tapering method, and a coating consisting of SiO_2_ nanoparticles, the OFHS developed in [72] and shown in Figure 11 offers a sensitivity of 584.2 pm/%RH. 

#### 2.4.4. Complex Refractive Index Materials Coated onto Modal Interferometers

An interesting study is the performance of modal interferometers, such as SMS structures, when they are coated with a material having a complex RI. As has been shown, the sensitivity to the external RI can be increased by coating the multimode segment [70,73]. The studies performed in [74,75] focused on coatings with materials having a complex RI and on the optimization of the coating parameters for achieving the maximum sensitivity. The most important conclusion extracted from [74] is that there is an optimum thickness of the coating that leads to a higher sensitivity to the thin-film refractive index, thin-film thickness, and surrounding medium refractive index. When the thickness of the coating approaches this optimum value, there is an attenuation of the observable interference pattern. This is the so-called fading region. This fading region is caused by the attenuation bands produced by the material having a complex RI. These attenuation bands are obtained at certain thicknesses of the coating and they experience a wavelength shift as the thickness increases. That is the reason for the interference pattern disappearing and appearing again. A similar study has been performed with photonic crystal fibers (PCF) [56], which behave following the same trend. Therefore, the fade region indicates the optimum thickness for obtaining the highest sensitivity.

### 2.5. Fabry-Pérot Cavities

One optical phenomenon extensively used for the development of optical fiber sensors and specifically for OFHSs fabrication is Fabry-Pérot interferometer (FPI). FPIs are based on the interference caused by multiple reflections of light between two reflecting surfaces. Transmitted beams, being in phase, generate constructive interference, whereas whether the transmitted beams are out of phase or not, destructive interference occurs and this corresponds to a transmission minimum. Constructive interference corresponds to a high-transmission peak of the etalon. Whether the multiply reflected beams are in phase or not depends on the wavelength of the light, the angle at which the light travels through the etalon, the thickness of the etalon, and the refractive index of the material between the reflecting surfaces [76]. This section focuses only on FPIs developed by coating the end facet of the optical fiber [77]. 

In such FPIs the two semi-reflective surfaces are generated by the fiber–coating interface and by the coating-air interface. Transparent conducting oxides have been proven a good choice for this optical structure, due to their optical constants and especially for humidity sensing purposes. The good performance of metal oxides as humidity transducers is due to the electrostatic attraction between the oxygen of the water molecule and the cationic side of the metal oxide surface, caused by the polar nature of the water molecule [78]. 

**Figure 12:**
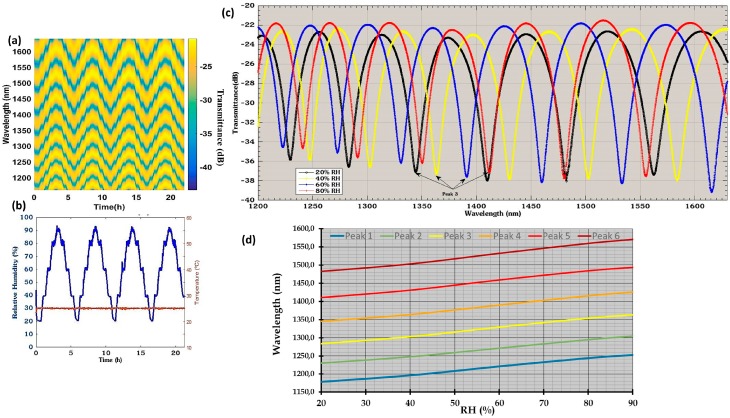
(**a**) Evolution of the reflected spectra for (**b**) several cycles of changing RH. (**c**) Reflected spectra for different values of RH and (**d**) wavelength of interference plotted as a function of RH. Reprinted from [77] with permission of Springer International Publishing.

Different materials have been studied, such as tin dioxide coated onto the end facet of a SMF [77], which presents a large dynamical range (90 nm), low response time, and a sensitivity of 1.27 nm/%RH. The optical response of tin dioxide to RH shows high linearity and low hysteresis, making it a good option for the development of OFHSs. Experimental results for this optical device are depicted in Figure 12.

Other humidity-sensitive materials used in FPIs include semiconductors [77,79], ceramics [80], polymers [81,82], etc. Porous anodic alumina has been studied and a sensitivity of 0.31 nm/%RH was achieved [80]. An important factor that must be taken into account when working with porous materials is the required time for complete desorption of water, which can range from a few seconds [82] to 22 minutes [80], depending on the size of the pore, among other factors.

A different approach is followed when the FPI is generated by a water-swelling material. Previous FPIs were based on changes in the RI of the material due to the water absorbed into the pores [80] and/or onto the metal oxide surface [77,79]. Water-swelling materials change their dimensions when they are subjected to changes of RH. Therefore, the length of the etalon is modified and subsequently the interference condition changes. This is the working principle of the FPI developed in [81], which is developed with a 10-µm Nafion film and has a sensitivity of 3.5 nm/%RH. This is the highest sensitivity found in this review.

### 2.6. Sagnac Interferometers

**Figure 13:**
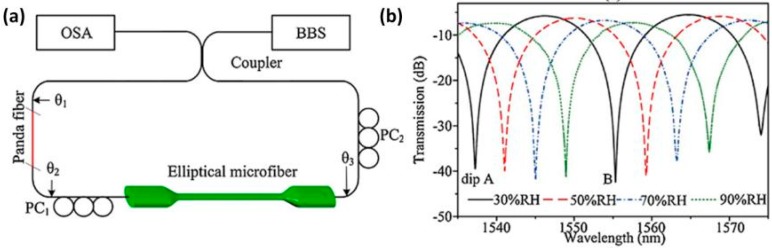
(**a**) Schematic diagram of the Hi-Bi elliptical microfiber Sagnac interferometer-based RH sensor; (**b**) Recorded transmission spectrum of dips at different RH values. Reprinted from [87] under creative common license https://creativecommons.org/licenses/by-nc-nd/4.0/.

The most well-known application for Sagnac interferometers is as a gyroscope [83], but they have also been widely studied and applied in other sensor applications [84]. Sagnac interferometers use a coherent monochromatic light source. Monochromatic light makes interfering behavior more predictable and coherence is required for phase shift detection [85]. The laser beam is split into two different beams forced to follow a single path but in opposite directions. After rounding the enclosed area, the two beams are recombined in the splitter and the phase shift becomes an optical power output variation. Two different approaches have recently been proposed [86]. Both of them use a similar setup (see Figure 13), which includes a broadband light source (BBS) and an optical spectrum analyzer (OSA). The main difference is related to the use of an additional sensitive layer in one case [86], whereas in the other case the sensing principle is based on the interaction between the evanescent field of a high birefringence (Hi-Bi) optical fiber [87] and the humidity of the environment.

Polyvinyl alcohol (PVA) is used as the sensitive material in the first approach [86]. It was coated onto a chemical etched polarization maintaining optical fiber (PMF), which forms one of the arms of the interferometer. The other arm is formed by an unmodified PMF. Therefore, interference will happen owing to the relative-phase difference introduced to the guided modes by the PMFs. The obtained attenuation bands, with attenuations of 20 and 25 dB, have a FWHM of 11.8 and 5.2 nm, respectively. A sensitivity of 111.5 pm/%RH was achieved within the humidity range 20–80%RH with a response time of about six seconds. Moreover, the sensitivity to temperature is only 7.2 pm/°C.

Greater sensitivities were obtained in [87]. The fabrication of this Sagnac interferometer, represented in Figure 13, requires a more complex setup for the fabrication of the sensitive optical fiber, which is a Hi-Bi optical fiber that consists of an elliptical microfiber. Furthermore, polarization controllers were needed. The final device shows a sensitivity of 422 pm/%RH with response times of only 60 ms. The ability of measuring RH without adding any coating enables faster measurements.

### 2.7. Resonators and Whispering Galleries Modes

The optical devices explained in this section are based on microtapers. In the first subsection, the microtapers constitute the resonator, while in the second subsection they are used to couple the light to the resonator structure. 

#### 2.7.1. Microloop and Microknot Resonators

This optical structure presents a desired characteristic for all optical sensors: their high *Q* factor. The *Q* factor is related to the quality of the filter performance and involves higher resolution for a sensor. Resonators can be fabricated in different ways.

In microloop resonators, due to the diameter of the fiber, a large fraction of the guided field is left outside the fiber as evanescent waves. Then, these evanescent waves can be self-coupled to the parallel segment of the microfiber and interfere with the light being guided through the loop. A microfiber loop resonator developed using standard SMF [88] achieved a sensitivity of 1.8 pm/%RH without any sensitive coating. Its Free Spectral Range (FSR) is 350 pm and the fringe contrast reaches 7 dB.

**Figure 14:**
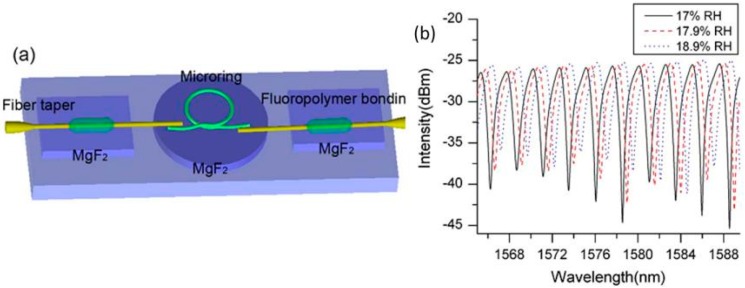
(**a**) Schematic diagram of a PAM microring for humidity sensing; (**b**) Redshift of resonance peaks when the microring is exposed to 17%RH, 17.9%RH, and 18.9%RH, respectively. Reprinted from [90] with permission from OSA Publishing.

A similar structure is the so-called microknot resonator. Two different microknot resonators (MKR) were developed in [89]. One of them was made of silica (standard SMF) and the other one was built using polymethyl methacrylate (PMMA) as the waveguide. The silica MKR (1.2 µm diameter) had a Q factor of 15,000 and a FSR of 0.22 nm. The PMMA MKR (2.1 µm diameter) showed a Q factor of 20,000 and a FSR of 0.17 nm. This last device has reached sensitivities of 8.8 pm/%RH (~8 times higher than the silica MKR) with a high resolution of 0.0023 RH. The greater sensitivity found for this optical structure is 490 pm/%RH [90] for a microknot developed with polyacrylamide (PAM), which is depicted in Figure 14.

#### 2.7.2. Whispering Galleries Modes 

This kind of resonator consists of two optical structures, the waveguide and the coupler. The dielectric resonator, with circular structures, supports the electromagnetic surface oscillations, which are evanescently coupled to the waveguide. Total internal reflections from the resonator’s curved surface confine the energy of the light inside the resonator, generating some transmission dips. The spectral positions of the transmission dips are strongly dependent on the geometry of the resonator and the optical properties of the resonator material. They offer high resolution and the ability to measure really low RH values.

**Figure 15:**
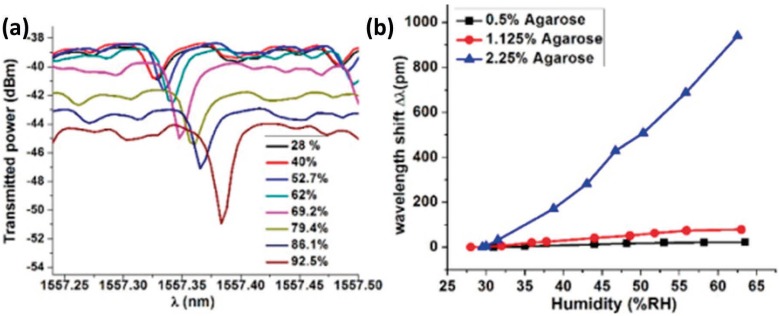
(**a**) Humidity response of the WGM spectrum for an uncoated microsphere resonator; (**b**) spectral shift versus RH for the sensors based on the same diameter microsphere coated with agarose solutions of different concentrations. Reprinted from [92] with permission from OSA Publishing.

A tapered optical fiber was used as the waveguide and a silica microsphere was used as the resonator structure [91,92]. The resonator was coated with SiO_2_ nanoparticles by layer-by-layer nano-assembly. This OFHS has a resolution of 0.003%RH [91] using the coated WGM microspheres at low RH (0%–12%RH). The other material tested using a similar structure was agarose, which provides a sensitivity of 518 pm/%RH [92]; the obtained results are shown in Figure 15.

**Figure 16:**
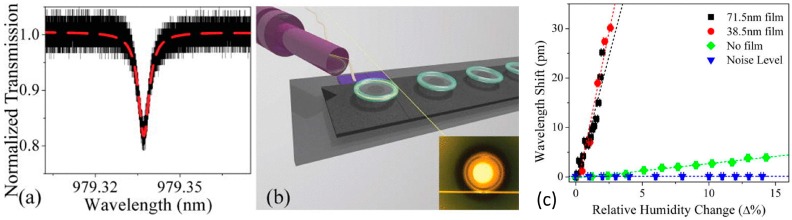
(**a**) A representative transmission spectrum for a thinner polymer-coated device while on resonance. The dashed line is a Lorentzian fit to the spectrum, which yields a Q of 2.5 × 10^5^; (**b**) Rendering of the testing setup. The inset shows the tapered optical fiber and the hybrid microtoroid; (**c**) Noise measurement and resonant wavelength shift as a function of relative humidity change at 23 °C for bare silica, thinner, and thicker polymer-film-coated devices. The error bars in the measurement are smaller than the symbols. Reprinted from [93] with permission of AIP Publishing LLC.

Finally, a similar structure but with toroidal form was used in [93] and is shown in Figure 16, achieving a sensitivity of 12.98 pm/%RH in the range 0%–12%RH. The silica microtoroid was coated with poly(N-isopropylacrylamide), which improves the sensitivity of the device by nearly two orders of magnitude. The Q-factor was 2 × 10^5^, providing high resolution.

## 3. Optical Fiber Humidity Sensors Based on Lossy Mode Resonances

The first time dealing with lossy mode resonances (LMR), these were confused with surface plasmon resonance (SPR) [94], but, despite its similarities, important differences can be distinguished between the phenomena. What makes them similar is that both are electromagnetic resonances that generate an attenuation band on the transmitted spectrum. However, in SPRs there is energy transference from light to free electrons of the noble metal, whereas in LMRs the light is coupled with the coating. Another difference is the possibility of being observed on the transverse magnetic (TM) and transverse electric (TE) modes, which might simplify the required setup and number of materials available for LMR generation, which broadens the application of LMR-based sensors. The possibility of generating several attenuation bands at tunable wavelengths is another advantage of LMRs. However, one of the most relevant factors that make LMRs a good choice for optical fiber sensors development is their ability to generate an optical phenomenon that can be detected by the wavelength detection method with the same material that acts as the sensitive layer to the parameter to be measured. 

**Figure 17:**
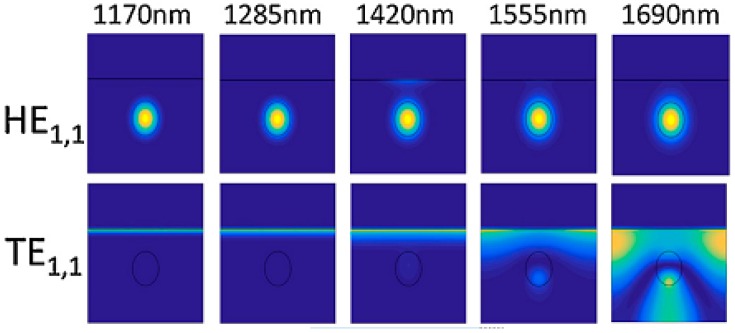
Electric field intensity in the transversal section of D-shaped optical fiber coated with ITO. The fundamental mode HE_1,1_ and the first mode that experiences a transition to guidance in the overlay (TE_1,1_), are analyzed for different wavelengths. Reprinted from [96] with permission from Elsevier.

The structure of a LMR-based device consists of a waveguide, which allows for accessing the evanescent field, coated with a thin film of the appropriate material. The condition for LMR generation is that the real part of the thin film permittivity is positive and higher in magnitude than both its own imaginary part and the real part of the material surrounding the thin film [95]. LMRs are generated when there is a resonant coupling of light to modes guided in the external coating. Figure 17 shows the fundamental mode (HE_1,1_) confined in the fiber core except at 1420 nm, where a fraction of the power transmitted by the core mode is coupled to the thin film, generating the LMR. It also shows one cladding mode (TE_1,1_) that is confined at the cladding at 1690 nm, but is guided through the coating for wavelengths smaller than 1420 nm [96]. 

Several materials, including metal oxides such as ITO [97], SnO_2_ [98,99], In_2_O_3_ [100], TiO_2_ [95] or polymers such as poly(allylamine hydrochloride) (PAH) and poly(acrylic acid) (PAA) [101], have been tested and checked for LMR generation. The large amount of available materials enables the development of optical fiber sensors for a wide range of applications [63,102,103,104,105]. Besides the research on the appropriate materials, LMR supporting structures have evolved and they have been studied on a wide range of optical fibers.

**Figure 18:**
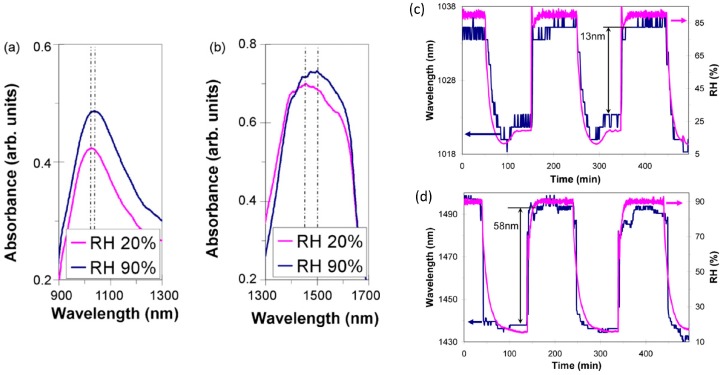
Spectral response of the (**a**) non-tuned and (**b**) tuned sensors for 20% and 90%RH. Dynamical response of the sensors to changes in the RH of the external medium (**c**) non-tuned sensor and (**d**) tuned sensor (non-tuned sensor (20 PAH/PAA bilayers) and tuned sensor (100 PAH/PAA bilayers)). Reprinted from [97] with permission from Elsevier.

The first LMR-based devices were developed on plastic-clad silica (PCS) optical fiber (200 µm diameter). Then, by chemically removing the cladding the evanescent field becomes accessible, which is one of the requirements for LMR generation. ITO was the first material tested and was coated by dip-coating [102], but other metallic oxides have been studied, such as tin dioxide [106]. The results obtained by this combination (LMR generated by tin dioxide and PCS) show a sensitivity of 0.1 nm/RH% in the range from 20% to 80%RH [106]. Adding an external layer, which is sensitive to humidity, the sensitivity of the device improves, reaching values around 1 nm/%RH [97]. Characterization of this last device is shown in Figure 18.

A method to decrease the resonance width and increase the sensitivity of LMR-based sensors is currently being studied [96,99,107]. Side polished optical fibers enable distinguishing between the TM and the TE modes of the LMR [108], obtaining a narrower attenuation band. Moreover, working with the fundamental mode provides better sensitivity than working with several modes [96,108,109]. 

Therefore, using a SMF improves the previously obtained sensitivities. Different approaches can be followed when working with SMF such as using side polished optical fibers, tapered optical fibers, or cladding etched SMF (CE-SMF). The first two methods have been used for the development of several kinds of sensors [63,101]. The latter has been recently studied towards humidity sensing [99]. Etching the cladding of a SMF with hydrofluoric acid (HF) reduces the cladding diameter, allowing the interaction with the evanescent field. 

**Figure 19:**
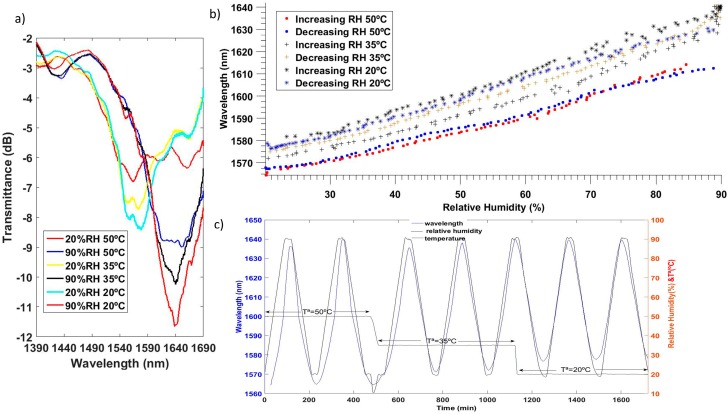
(**a**) Optical spectrum of the SnO_2_ coated CE-SMF for different RH values; (**b**) characterization of the LMR wavelength as a function of RH for different temperatures; (**c**) LMR wavelength and RH as a function of time for different temperatures. Reprinted from [99] with permission from Elsevier.

Two different materials have been tested with CE-SMF structure, indium oxide [100] and tin oxide [99]. Both transparent conductive oxides meet the requirements for LMR generation. The real part of the refractive index of tin oxide is slightly greater, increasing the sensitivity of the final device [99]. The wavelength of the LMR shifts more than 30 nm for RH changes from 20% to 90% when working with indium oxide. The LMR generated by tin oxide has a sensitivity of 1.9 nm/%RH for the same range of RH. Static and dynamic characterization of this sensor is plotted in Figure 19. In addition, this optical structure has an unnoticeable cross thermal sensitivity. High linearity and low hysteresis are other characteristics of this kind of sensor.

An interesting comparison between SMS and LMR was developed in [110] by coating a SMS and a PCS (200 µm diameter) with a combination of titanium (IV) oxide nanoparticles (TiO_2_) and poly(sodium 4-styrenesulfonate) (PSS). When comparing the sensitivity to relative humidity changes, the LMR-based device shows a tenfold improvement compared to the SMS-based device. Therefore, it is proved that it is better to use an LMR-based device if high sensitivity is needed. However, the SMS structure provides narrower attenuation bands than LMR generated onto PCS fibers. In any case, coating the SMS structure with the thin film provides better sensitivity than the naked SMS.

LMRs can be combined with another optical phenomenon such as Localized Surface Plasmon Resonance (LSPR). This was demonstrated by using the Layer-by-Layer nano-assembly (LbL) method to develop a polymeric coating loaded with Ag nanoparticles (Ag NPs) onto an optical fiber [111]. The LSPR band showed a slight intensity variation with RH changes but no significant wavelength dependence was observed. Therefore, the LSPR band can be used as a reference wavelength. Furthermore, the polymeric coating with silver nanoparticles is twice as sensitive (0.943 nm per %RH) to RH changes than the polymeric overlay only (0.44 nm per %RH).

This paper has tried to encompass the most recent and relevant research papers involving optical fiber humidity sensors. The main optical structures used for that purpose were briefly explained, intending to provide a complete guide of recent developments in this application field. Table 1 summarizes some of the most relevant results.

## 4. Conclusions

There are several ways to measure relative humidity by optical setups and by OFHS and several application fields where this kind of sensor can be exploited, such as radiation environments or watertight containers, or for example vacuum-packed foods, where other sensors might alter the environment and distort the measurement. Although there are structures that have not been analyzed, some interesting conclusions can be extracted from those that have been explained here.

Novel materials, understood as novel nanostructured materials (nanotubes, quantum dot), are still appearing and show promising properties for humidity sensing. Polymeric coatings and inorganic salts might present nonlinear behavior, especially at high relative humidity values, where they have their greatest sensitivity. Metal oxides and semiconductor oxides seem to be a good choice for obtaining linear responses and good sensitivity in the 20%–90%RH range. Their response times are usually shorter than those of water-swelling materials.

With regard to optical structures, there should be an agreement between the desired spectral width of the attenuation band and its dynamic range. Another important factor that must be taken into account is the complexity of the required fabrication process. Modal interferometers developed by splicing together different kind of fibers represent the largest number of publications in recent years. In addition, polymeric optical fibers and photonic crystal fibers have shown their strength for optical fiber sensor development.

FBGs and LPFGs require specific equipment for inscribing the grating onto the optical fiber. They are not too sensitive to external parameters by themselves and need an external coating besides the grating. However, recent research has demonstrated that improved sensitivities can be obtained with these optical structures. Moreover, they compensate for their small dynamical range with their high resolution.

For LMRs, a previous process is required to access the evanescent field. This process is simpler than that required for writing a grating and usually consists of a chemical method to partially or completely remove the cladding. LMRs allow for generating an attenuation band with the same material that will act as a sensitive layer, simplifying the process of obtaining an optical fiber humidity sensor. 

Finally, Fabry-Pérot interferometers, developed by coating the tip of an optical fiber, seem to be a good choice to obtain optical fiber humidity sensors because of their good performance and the relative ease of obtaining them. They are also a less invasive way to measure with optical fibers. The highest sensitivity found in this review corresponds to this optical structure dealing with hygroscopic materials.

## Figures and Tables

**Table 1:** Summary of the most relevant results found for the interval 2010–2016.

Reference	Year	Method	Sensing Material	Range	Sensitivity/Resolution	Response Time	Comments
***Optical absorption sensors***							
[16]	2016	D-shape SMF	WS_2_	35–85%	0.123 dB/%RH & 0.475 %RH	1–5 s	-
[17]	2014	D-shape SMF	rGO	75–95%	0.31 dB/%RH	0.13%RH/s	-
***Fiber Bragg gratings***							
[31]	2015	Δλ_B_,strain	Polyimide	11–97%	13.6pm/%RH	22–29 min	*In situ imidization*
[33]	2014	Etched FBG, RI	CNT	20–90%	31 pm/%RH & 0.03%RH	9.7–39.4 min	*-*
[27]	2016	Bragg on POF	PMMA	10–90%	35 pm/%RH		Annealed 90%RH
***Long-period fiber gratings***							
[37]	2014	RI, λ	TiO_2_	0–75%RH	1.4 nm/%RH at low RH	-	Radiation&T^a^<0°C
[45]	2010	RI, λ	Calcium chloride	55–90%RH	1.36 nm/%RH	-	Air gap LPG
[46]	2015	RI, swelling	PAH/PAA	20–80%RH&25–85°C	63 pm/%RH & 411pm/°C	-	RH & Tª
***Interferometers***							
***Fabry–Pérot***							
[77]	2015	RI, absorption	SnO_2_	20–90%RH	1.26nm/%RH & 0.06%RH	-	-
[81]	2014	Swelling, RI	Nafion	22–80%RH	3.5 nm%RH & 3 × 10^−4^%RH	242 ms (ΔRH=3%)	
***Sagnac***							
[87]	2016	Elliptical microtaper	No coating	30–90%RH	422 pm/%RH	60 ms	
[86]	2013	Etched PMF	PVA	20–80%RH	111.5 pm/%RH	6s	
***Modal Interferometers***							
[56]	2016	Photonic crystal fiber	PAH/PAA	20–75%RH 75–95%RH	0.29 nm%RH 2.35 nm/%RH	200 ms	-
[64]	2013	Tapered optical fiber	No coating	30–90%RH	97.8 pm/%RH	188 ms	-
[72]	2016	SMS	SiO_2_ nanoparticles	44–98.6%RH	584.2 pm/%RH	-	-
***Resonators***							
[88]	2013	Microloop	No coating	50–80%RH	1.8 pm/%RH	-	-
[89]	2010	Microknot	Silica or PMMA microfiber	20–96%RH 17–98%RH	1.2 pm/%RH 8.8 pm/%RH	<0.5 s	-
[93]	2013	Microtoroid+tapers	poly(N-isopropylacrylamide)	0–60%RH	13 pm/%RH	1.6 s<t<5 s	Q-factor
***Lossy Mode Resonances***							
[106]	2013	Dip-coating (PCS 200 µm)	SnO_2_	20–90%RH	0.1 nm/%RH	-	-
[102]	2012	LbL onto PCS 200 µm	In_2_O_3_+PAH/PAA	20–80%RH	0.935 nm/%RH	-	-
[99]	2016	Sputtering onto CE-SMF	SnO_2_	20–90%RH	1.9 nm/%RH	1.5–4 s	-
